# Physicochemical, Nutritional, and Antioxidant Properties in Seven Sweet Potato Flours

**DOI:** 10.3389/fnut.2022.923257

**Published:** 2022-06-15

**Authors:** Lin Zhang, Yan Gao, Bowen Deng, Weidong Ru, Chuan Tong, Jinsong Bao

**Affiliations:** ^1^Hainan Institute, Zhejiang University, Yazhou Bay Science and Technology City, Sanya, China; ^2^Institute of Nuclear Agricultural Sciences, Key Laboratory of Nuclear Agricultural Sciences of Ministry of Agriculture and Zhejiang Province, College of Agriculture and Biotechnology, Zhejiang University, Hangzhou, China; ^3^College of Food Science and Technology, Zhejiang University of Technology, Hangzhou, China

**Keywords:** sweet potato, wholemeal flour, nutritional properties, physicochemical properties, phenolics

## Abstract

Sweet potato flour is a key ingredient for the production of new food products worldwide, which imparts desired properties, nutritional value, antioxidants, and natural color to processed foods. However, little information regarding the functional properties of the sweet potato flour is available. In this study, the genetic diversity in the physiochemical, nutritional, and antioxidant properties of wholemeal flour from seven sweet potato varieties was investigated. The total phenolic content (TPC) of the free and bound fractions ranged from 13.85 to 90.74 mg gallic acid equivalent (GAE)/100 g and from 5.07 to 24.29 mg GAE/100 g, respectively. The average protein content of sweet potato was 5.41 g/100 g ranging from 3.40 to 8.60 g/100 g DW. The total amino acid content averaged 45.13 mg/g DW. The average contents of 12 mineral elements were in the order of K > P > Ca > Mg > Mn > Fe > Zn > Cu > Ni > Se > Cr > Cd. K and P contents were the highest among all accessions, which were positively correlated with most of the other minerals. The average starch content of sweet potato was 53.90 g/100 g DW, ranging from 31.68 to 64.90%. The peak viscosity (PV), hot paste viscosity (HPV), and cold paste viscosity (CPV) were in the range of 90.7–318.8 Rapid Visco Unit (RVU), 77.3–208.3 RVU, and 102.6–272.7 RVU, respectively. The hardness values and cohesiveness (Coh) varied among different sweet potatoes, with a range of 8.20–18.48 *g* and 0.22–0.68, respectively. The gelatinization onset, peak, conclusion temperatures, and enthalpy were in the ranges of 59.39–71.91°C, 70.19–88.40°C, 78.98–95.79°C, 1.85–5.65 J/g, respectively.

## Introduction

Sweet potato (*Ipomoea batatas* L.) as a tuberous root belongs to the Convolvulaceae family, is seventh in the world’s crop statistics (after wheat, rice, corn, potatoes, cassava, and barley) (1), and is widely grown in more than 100 countries of tropical, subtropical, and temperate regions. The worldwide planting area and the total annual output of sweet potato reached 7.7689 × 10^6^ hm^2^ and 9.1821 × 10^7^ t in 2019 (2). Sweet potato is highly rich in starch, dietary fiber, minerals, vitamins, and phytochemicals with antioxidant activities, such as ascorbic acid, carotenoids, flavonoids, and other phenolic compounds (3, 4). Significant variation in nutrient composition, such as protein, β-carotene, calcium, magnesium, phosphorus, and potassium, was found among the sweet potato varieties (5). Abegunde et al. (6) found the flours and starches from different sweet potato cultivars differed in their amylose, amylopectin, ash and phosphorus contents, starch granule size, water absorption and solubility, retrogradation, crystallinity, and their thermal and pasting properties. In general, white-fleshed sweet potato had a high percentage of carbohydrate and reduced sugar and phenolics, and purple-fleshed sweet potato had high anthocyanin contents and antioxidant capacities, while yellow- and orange-fleshed ones had high levels of total protein, flavonoids, anthocyanins, and carotenoids (7).

The functional properties of sweet potato flours play an essential role in food manufacturing. Such properties decide the production and use of sweet potato flours as food ingredients for different foods and also regulate the processing and storage of these items (8). For example, functional properties, such as water absorption, oil absorption, and protein solubility, affect the product’s texture and appearance (9). Sajeev et al. (10) studied the textural and gelatinization characteristics of white sweet potato, cream, and orange sweet potato flours and they indicated that the considerable mealiness found in the white fleshed colored tubers could be a good option for infant food formulations. In their research with flours from 25 sweet potato cultivars, they reported that cultivars with restricted swelling and gelatinization might not be suitable for baked goods, where the final product volume is a quality parameter (10).

Mostly, sweet potatoes in China are consumed as fresh food or feed, and a few sweet potatoes are processed into starch or flour for vermicelli production (11). Processing and conversion of sweet potatoes into a staple form is also suggested as a choice to address the storage and transportation problems of fresh sweet potatoes. Processing of sweet potato not only increases the income of farmers and processors (12, 13) but also raises the awareness of the utilization potential of this crop worldwide (13, 14). However, little information about the genetic diversity in the physicochemical, antioxidant, and nutritional properties of wholemeal sweet potato flour is available.

In this study, seven sweet potato varieties were used to analyze the biodiversity in nutritional components and physicochemical properties of sweet potato wholemeal flour. The results of this study will provide a theoretical basis for the selection of suitable potato whole flour for food processing.

## Materials and Methods

### Materials

Seven pigmented sweet potato varieties (SP01 “Zheshu75,” SP02 “Zheshu 6025,” SP03 “Zheshu33,” SP04 “Zheshu 13,” SP05 “Zheshu 132,” SP06 “Zheshu 81,” and SP07 “Zheshu 259”) with different genetic background were used in this study. They were planted in early June 2017 in Anji, Zhejiang Province, China, and the root tubers were harvested in later October 2017. Harvested sweet potato roots of similar size were washed and peeled manually and were cut to pieces being before stored at –80°C. It is well known that different drying methods have different effects on the quality of sweet potato wholemeal flour (15–17). The freeze-drying method was applied to minimize the negative effects of the drying method on the physicochemical properties. The frozen sweet potato pieces were freeze dried for 48 h (vacuum freeze drier, Model FD-1A-135, Beijing, China). The dried sweet potato samples were ground into flour and stored at 4°C until the next analysis.

### Extraction of Free/Soluble and Bound Phenolics

The extraction method of free/soluble phenolics was performed according to Ru et al. (18). Sweet potato flour (1.0 g) was defatted with 10 ml hexanes before to times extraction with 20 ml of 80% methanol at room temperature. The mixture solution was shaken for 30 min before centrifugation at 8,000 × *g* for 5 min. The supernatant was collected and adjusted its pH to 1.5–2.0 before being concentrated at 37°C using a rotary evaporator (IKA RV 10 digital V, Staufen, Germany). The concentrated solution was extracted with 20 ml ethyl acetate in triplicate. Each extract was combined and then evaporated using a rotary evaporator at 35°C. Finally, the evaporated extract was dissolved with 5 ml of 50% methanol, and the free phenolic acid solution was stored in the refrigerator at −20°C until the next analysis.

The solid residue after extracting soluble phenolics was subsequently used to extract bound phenolics. The residue was firstly digested with 20 ml of NaOH (4 M) for 2 h at room temperature. The mixture solution was adjusted to pH 1.5–2.0 using HCl before being extracted three times using 60 ml ethyl acetate. The pooled ethyl acetate extractions were evaporated and dissolved in methanol followed the same extraction procedures of free soluble phenolics.

### Total Phenolic Content (TPC)

The TPC was measured using the colorimetric method with Folin–Ciocalteu reagent as described elsewhere (18). The TPC was expressed as milligrams of gallic acid equivalent (mg GAE) per 100 g of sweet potato.

### Antioxidant Activity

1,1-diphenyl-2-picrylhydrazyl radical 2,2-diphenyl-1-(2,4,6-trinitrophenyl) hydrazyl (DPPH)⋅ assay was accomplished with the description of Ru et al. (18) with minor modification. Briefly, 200 μL of the diluted free or bound phenolics extracts were added to 3 mL OF DPPH⋅ radical solution (100 μM) with methanol. The reaction was kept in the dark at room temperature for 30 min, before measuring its absorbance at 517 nm. DPPH⋅ scavenging activity was expressed with inhibition (percent) of DPPH⋅ absorbance. The DPPH scavenging activity (%) of both samples and standard (Trolox) was calculated as follows: DPPH% = (1 – A_sample_/A_control_) × 100%.

1,1-diphenyl-2-picrylhydrazyl radical 2,2-diphenyl-1-(2,4,6-trinitrophenyl) hydrazyl radical scavenging activities of crude extracts were expressed as μM of Trolox equivalents (TE) per gram of sweet potato flour using a standard curve of Trolox.

The assay of 2,2-azino-bis-3-ethylbenzothiazoline-6-sulphonic acid diammonium salt (ABTS) + radical cation scavenging activity was conducted according to the method described in Ru et al. (18). Firstly, 7 mM of ABTS and 2.45 mM of potassium per sulfate were mixed at room temperature in dark for 20 h to generate ABTS^+^ radical cation. Then, methanol was used to dilute the ABTS^+^ mixture to an absorbance around 0.700 at 734 nm. Finally, 3.9 ml of ABTS + solution was added to 0.1 ml of extracts. The reaction mixture was kept at room temperature for 6 min, then the absorbance was recorded at 734 nm. Trolox (0.5 mM) was served as a reference antioxidant.

### Starch and Protein Content

The content of total starch was determined with an acid hydrolysis method (GB 5009.9–2016). Briefly, about 2 g of sweet potato flour was weighed and placed into a slow filter paper funnel. In total, 50 ml of petroleum ether was added to remove lipids, before moving soluble sugars with 150 ml 85% ethanol. The filter residue was placed in a 250 ml flask, added 30 ml of hydrochloric acid and supplemented with distilled water to 100 ml, inserted into a condensing tube, refluxed in a boiling water bath for 2 h, cooled to room temperature, neutralized with sodium hydroxide solution until slightly acidic, and filtered through qualitative filter paper or skim cotton in a 500 ml of volumetric flask. The reducing sugar content of the reserve solution was determined by using Fehling’s solution and then was converted to starch content. Protein content was carried out according to the Kjeldahl method with Kjeltec-Foss 2400 Auto-Analyzer (Foss, Denmark). The conversion coefficient of nitrogen to protein was 6.25.

### Amino Acid Content

Extraction of sweet potato amino acid was followed by the method of Xu et al. (19). Free amino acids were extracted from 0.5 g flour samples with 300 ml 3% sulfosalicylic acid by vigorously shaking for 1 h. The suspension was then centrifuged at 10,000 × *g* for 10 min at 4°C. After three such extractions, the extracts were combined, filtered with 0.45 μm syringe filters, and stored at 4°C for analysis using the ninhydrin method with an automatic amino acid analyzer (L-8900, Hitachi, Tokyo, Japan).

### Mineral Content

Sweet potato flour (0.5 g) was placed into a graduated polyethylene vial. The concentrated nitric acid (5.0 ml) and H_2_O_2_ (1.0 ml) were added before the polyethylene watch glass was covered and stood overnight. Samples were digested at a maximum temperature of 130°C until the solution became clear. The digests were diluted with deionized H_2_O to 50.0 ml. The minerals in the diluted solution were analyzed by an ICAP 6000 inductively coupled plasma optical emission spectrometer (ICP-OES) (Thermo Scientific, Cambridge, United Kingdom).

### Pasting Properties

The viscosity characteristics of sweet potato flour were tested with an RVA instrument (RVA, Model 4500, Perten Instruments, Hägersten, Sweden). In total, 3.0 g of sweet potato flour and 25 ml of double deionized H_2_O were added into the aluminum can and were mixed well. Peak viscosity (PV), hot paste viscosity (HPV), cold paste viscosity (CPV), and two derivative parameters, i.e., breakdown (BD = PV − HPV) and setback (SB = CPV − HPV), were obtained. The viscosity was measured in Rapid Visco Units (RVU).

### Texture Properties

After testing the RVA of the whole sweet potato powder, the aluminum sample tube was sealed with Parafilm ™ and placed in a refrigerator at 4°C for 24 h. Then the TA-XT2i texture analyzer (Stable Micro Systems, Surrey, United Kingdom) was used to determine the texture characteristics of the whole sweet potato flour. The texture analyzer uses a probe with a diameter of 5 mm to perform the test repeatedly four times, and the program was set to the displacement distance of 10 mm and the probe descent speed of 1 mm/s (20). In the texture map, the highest peak of the curve is called the hardness (HD), and the ratio of the area of the second compression to the first compression is called the cohesiveness (Coh).

### Thermal Properties

The gelatinization properties of the whole sweet potato powder were measured using a differential scanning calorimeter (DSC-2920, TA Instruments, United States). First, weigh 2.0 mg whole sweet potato powder and mix it with 6 μl of ultrapure water in an aluminum pan, seal the aluminum pan, and let it stand at room temperature for 2 h. The differential scanning calorimeter program settings were as follows: the nitrogen flow rate is set to 50 ml/min, the temperature rises from 30 to 110°C, and the heating rate is 10°C/min. An empty pan was sealed as a reference sample. Parameters, such as onset temperature (To), peak temperature (Tp), conclusion temperature (Tc), and enthalpy of gelatinization (ΔH) were determined using the Universal Analysis (version 4.4A).

### Statistical Analysis

The results were presented as mean ± standard deviation (SD), in which all the measurements were accomplished at least in duplicate. Duncan’s multiple range test of ANOVA and correlation analysis were conducted using the SPSS 20.0 software (SPSS, Inc., Chicago, IL, United States).

## Results and Discussion

### Total Phenolic Content

Phenolic compounds and flavonoids are the most common phenolic compounds as free and bound forms in nature (21). The TPC of the free fraction was much higher than the bound fraction in sweet potatoes (Table 1). The TPC of the free and bound fractions ranged from 13.85 to 90.74 mg GAE/100 g and from 5.07 to 24.29 mg GAE/100 g, respectively. The TPC of free fraction in SP05 was the highest among these sweet potato samples. In numerical terms, the TPC levels of the free and bound fractions of sweet potato were the lowest in SP01 and SP07. The TPC levels of sweet potatoes in this study (21.25–104.40 mg GAE/100 g) were greatly lower than that of Virginia-grown sweet potatoes (140.00–1230.00 mg GAE/100 g) (22). The variations may be attributed to the variety and extraction method of TPC. Furthermore, the TPC content of sweet potato was generally based on the soluble phenolic acid extraction and ignoring the bound phenolic acids (23). Here, our results showed that the bound phenolic content could account for 13.08–37.87% of the TPC.

**TABLE 1:** TPC, DPPH, and ABTS radical scavenging of free and bound fractions of sweet potatoes.

Code	TPC		DPPH		ABTS	
	Bound	Free	Bound	Free	Bound	Free
SP01	7.40 ± 2.70^c^	13.85 ± 0.33^d^	16.83 ± 0.55^d^	56.14 ± 1.50^f^	11.80 ± 2.05^c^	38.14 ± 4.65e
SP02	9.76 ± 0.93b^c^	16.01 ± 0.11^cd^	16.28 ± 0.47^d^	65.82 ± 1.24^e^	10.87 ± 0.45^c^	41.64 ± 3.85e
SP03	6.67 ± 0.25^c^	23.21 ± 1.09^cd^	42.84 ± 7.39^c^	95.10 ± 1.59^d^	35.00 ± 11.82^b^	72.35 ± 0.40c
SP04	12.69 ± 2.19^b^	25.89 ± 0.14^c^	62.21 ± 2.28^b^	102.54 ± 1.76^c^	30.01 ± 8.75^bc^	56.88 ± 0.61d
SP05	13.66 ± 3.13^b^	90.74 ± 11.62a	43.02 ± 8.37^c^	284.55 ± 0.92^a^	19.99 ± 8.76^bc^	288.67 ± 7.40a
SP06	24.29 ± 2.44^a^	67.21 ± 4.06^b^	87.61 ± 6.55^a^	270.03 ± 1.27^b^	77.44 ± 11.41^a^	230.18 ± 3.70b
SP07	5.07 ± 1.76^c^	21.22 ± 0.12^cd^	6.54 ± 1.39^d^	95.98 ± 5.18^d^	12.26 ± 2.97^c^	67.77 ± 1.30c

*The results (means ± SD) of TPC are presented as mg gallic acid equivalent/100 g sweet potato powder, and the results (means ± SD) of DPPH and ABTS are presented as μmol Trolox equivalent/100 g sweet potato powder. The values in each column with different letters are significantly different (p < 0.05). TPC, total phenolic content. DPPH, 1,1-diphenyl-2-picrylhydrazyl radical 2,2-diphenyl-1-(2,4,6-trinitrophenyl) hydrazyl. ABTS, 2,2-azino-bis-3-ethylbenzothiazoline-6-sulphonic acid diammonium salt.*

### Antioxidant Capacity and Its Relations With Total Phenolic Content

*In vitro* antioxidant activity of sweet potato extracts was evaluated by DPPH⋅ and ABTS + free-radical scavenging capacities. Results showed that DPPH⋅ and ABTS + radical scavenging capacity of sweet potatoes differed significantly among varieties (Table 1). Among all the samples, the DPPH⋅ and ABTS + radical scavenging activities of the bound fraction were much lower than those of the free fraction. The DPPH⋅ radical scavenging activities of free and bound fractions ranged from 56.14 to 284.55 and from 6.54 to 87.61 μM TE/100 g, respectively. The ABTS + radical cation scavenging activity of free and bound fractions ranged from 38.14 to 288.64 and from 10.87 to 77.44 μM TE/100 g, respectively. Results showed for the free fraction that SP05 had a much higher antioxidant capacity and SP06 had a much higher antioxidant capacity of bound fraction.

Pearson correlations between the TPC of free and bound fractions and total antioxidant activities were analyzed to reveal the contribution of TPC to the antioxidant activity (Table 2). No matter what fractions, free or bound, the correlation coefficients between TPC, DPPH, and ABTS were higher than 0.83, indicating that the higher the phenolic content, the higher the antioxidant activity. The correlations between free and bound fractions were significant (*p* < 0.01). These findings were similar to the previous studies (22–24).

**TABLE 2:** Pearson pairwise correlations between TPC, DPPH, and ABTS in the free and bound fractions in the seven sweet potatoes.

		Free fraction			Bound fraction		
		TPC	DPPH	ABTS	TPC	DPPH	ABTS
Free fraction	TPC	1					
	DPPH	0.974**	1				
	ABTS	0.990**	0.988**	1			
Bound fraction	TPC	0.633*	0.748**	0.661*	1		
	DPPH	0.536*	0.644*	0.543*	0.857**	1	
	ABTS	0.419	0.582*	0.474	0.839**	0.898**	1

**indicate significant at p ≤ 0.01. *indicate significant at p ≤ 0.05.

### The Starch and Protein Content

**FIGURE 1:**
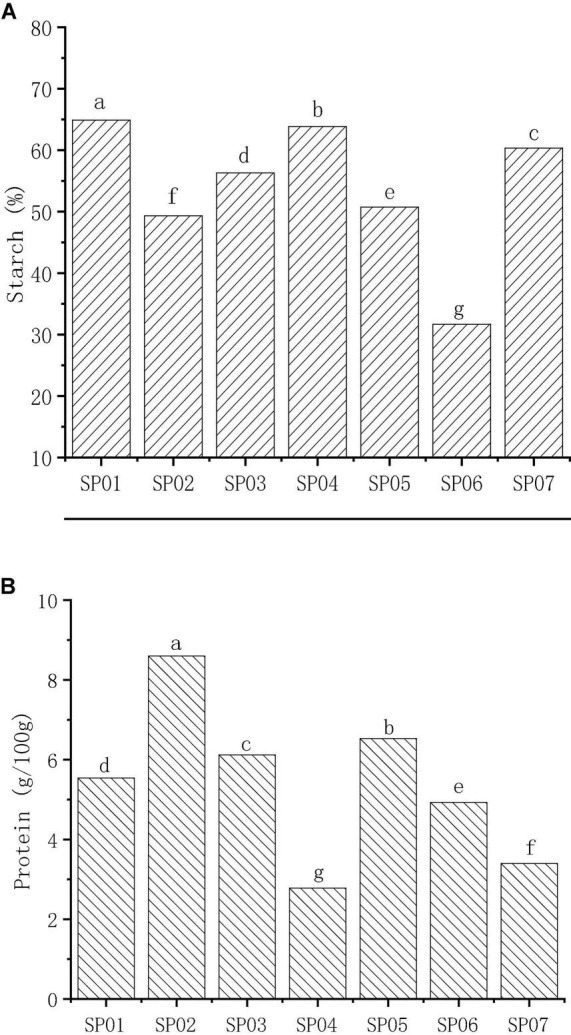
The content of starch **(A)** and protein **(B)** in different varieties of sweet potato powder. Different letters indicate significant differences (*p* < 0.05).

Starch was the principal carbohydrate in seven sweet potatoes with an average content of 53.90%, ranging from 31.68 to 64.90% (Figure 1A). The starch content of different sweet potato flours was quite different. The average starch content of sweet potatoes in this study was similar to those of Virginia-grown varieties (55.1% on average) (22). The starch content of SP01 was 64.90%, which was significantly higher than the other samples (*p* < 0.05) and was approximately two times higher than the values in SP06. These results were different from other studies in that total starch content varies between 57 and 90% (4), 55.76 and 83.65% (25), and 36.6 and 75.0% (26), due to different regions and varieties. In a previous study, Tong et al. (27) investigated the amylose content (AC) of the same set of sweet potato varieties used in this study and found that AC varied from 18.63 (SP02) to 20.45% (SP05).

The mean total protein content of sweet potato was 5.41 g/100 g DW with a range of 2.78–8.60 g/100 g DW (Figure 1B), which was consistent with the results of Osundahunsi et al. (28). The protein content in different sweet potato flours was 1.0–14.2% (4), 1.9–2.6% (29), and 2.4–2.9% (30), dependent on the environmental conditions. It is found that the total protein content differs significantly between different sweet potato varieties. SP02 had the highest content (8.60 mg/100 g), and SP04 had the lowest content (2.78 mg/100 g). This shows that among the seven kinds of sweet potato, SP02 is a high-protein variety.

### Amino Acid Contents

The content of 17 free amino acids and gamma-aminobutyric acid (GABA) in sweet potato flour was detected (Table 3). The average total amino acid content of seven sweet potato varieties was 45.13 mg/g, of which SP02 had the highest content (77.07 mg/g), and SP04 had the lowest content (20.44 mg/g). Hou et al. (31) reported free amino acids accounted for less than 3.1% of the total amino acids in corn kernels, peanuts, pistachios, soybeans, wheat, and white rice, but 34 and 28% in potatoes and sweet potatoes, respectively. This is inconsistent with our results and might be attributed to the different species selected. Aspartic acid (Asp) and glutamic acid (Glu) were the two most abundant amino acids in sweet potatoes (Table 3). The average content of GABA in these sweet potatoes was 0.19 mg/g, of which SP06 had the highest GABA content (0.25 mg/g), and the GABA content of SP02 was the lowest at 0.12 mg/g.

**TABLE 3:** Amino acid content of sweet potato powder with different varieties (mg/g).

	Asp	Ser	Glu	Gly	Ala	Cys	GABA	Lys	His	Arg	Pro	Thr	Val	Met	Ile	Leu	Tyr	Phe	Essential amino acid	Non-essential amino acid	Total
SP01	11.67 ± 0.60^c^	2.39 ± 0.13^d^	4.35 ± 0.32^d^	1.65 ± 0.05^e^	1.80 ± 0.05^e^	0.59 ± 0.02^a^	0.24 ± 0.11^a^	1.90 ± 0.12^e^	0.69 ± 0.05^e^	1.54 ± 0.16^e^	1.25 ± 0.04^d^	2.14 ± 0.12^c^	2.32 ± 0.07^d^	0.58 ± 0.05^d^	1.47 ± 0.05^d^	2.32 ± 0.10^e^	0.81 ± 0.02^a^	2.85 ± 0.02^cd^	12.49 ± 0.41^e^	28.05 ± 1.33^d^	40.54 ± 1.74^d^
SP02	20.17 ± 0.57^a^	5.05 ± 0.08^a^	8.56 ± 0.10^a^	3.32 ± 0.06^a^	3.84 ± 0.07^a^	0.57 ± 0.00^a^	0.12 ± 0.00^a^	3.85 ± 0.08^a^	1.42 ± 0.03^a^	3.39 ± 0.05^a^	2.70 ± 0.08^a^	4.21 ± 0.09^a^	4.75 ± 0.15^a^	1.23 ± 0.04^a^	3.08 ± 0.10^a^	5.10 ± 0.09^a^	1.14 ± 0.03^a^	4.58 ± 0.06^a^	24.09 ± 0.56^a^	52.98 ± 1.11^a^	77.07 ± 1.67^a^
SP03	15.14 ± 0.17^b^	3.53 ± 0.00^b^	6.53 ± 0.08^b^	2.62 ± 0.04^b^	3.09 ± 0.00^b^	0.66 ± 0.08^a^	0.19 ± 0.06^a^	3.15 ± 0.10^b^	1.12 ± 0.01^b^	2.46 ± 0.08^b^	2.20 ± 0.01^b^	3.14 ± 0.00^b^	3.34 ± 0.04^b^	0.79 ± 0.04^b^	2.14 ± 0.01^b^	3.89 ± 0.00^b^	0.80 ± 0.23^a^	3.45 ± 0.19^bc^	17.55 ± 0.51^b^	40.7 ± 0.29^b^	58.25 ± 0.80^b^
SP04	3.01 ± 0.34^g^	1.24 ± 0.02^f^	2.32 ± 0.21^f^	0.95 ± 0.00^g^	1.04 ± 0.06^g^	0.61 ± 0.09^a^	0.21 ± 0.03^a^	1.17 ± 0.04^g^	0.28 ± 0.02^g^	0.78 ± 0.01^g^	0.65 ± 0.03^f^	1.10 ± 0.07^e^	1.26 ± 0.05^f^	0.57 ± 0.05^d^	0.73 ± 0.03^f^	1.25 ± 0.02^g^	1.16 ± 0.12^a^	2.10 ± 0.15^e^	8.17 ± 0.49^e^	12.27 ± 0.2^e^	20.44 ± 0.69^f^
SP05	10.50 ± 0.64^d^	3.46 ± 0.07^b^	5.57 ± 0.19^c^	2.51 ± 0.04^c^	2.84 ± 0.07^c^	0.71 ± 0.05^a^	0.16 ± 0.04^a^	2.86 ± 0.08^c^	0.99 ± 0.00^c^	2.24 ± 0.11^c^	2.10 ± 0.08^b^	3.02 ± 0.11^b^	3.29 ± 0.10^b^	0.71 ± 0.08^bc^	2.09 ± 0.10^b^	3.65 ± 0.09^c^	0.90 ± 0.10a	3.65 ± 0.19^b^	17.31 ± 0.95^c^	33.93 ± 0.42^c^	51.24 ± 1.37^c^
SP06	7.19 ± 0.18^e^	2.72 ± 0.05^c^	5.15 ± 0.21^c^	2.08 ± 0.05^d^	2.43 ± 0.01^d^	0.64 ± 0.11^a^	0.25 ± 0.06^a^	2.58 ± 0.02^d^	0.78 ± 0.01^d^	1.86 ± 0.03^d^	1.73 ± 0.05^c^	2.31 ± 0.07^c^	2.60 ± 0.11^c^	0.62 ± 0.06^cd^	1.63 ± 0.05^c^	2.98 ± 0.05^d^	1.13 ± 0.24^a^	3.48 ± 0.37^bc^	14.75 ± 0.95^d^	27.4 ± 0.35^e^	42.15 ± 1.30^d^
SP07	4.51 ± 0.62^f^	1.71 ± 0.12^e^	2.92 ± 0.35^e^	1.27 ± 0.00^f^	1.38 ± 0.03^f^	0.70 ± 0.10^a^	0.18 ± 0.11^a^	1.57 ± 0.04^f^	0.41 ± 0.03^f^	1.17 ± 0.07^f^	0.95 ± 0.00^e^	1.56 ± 0.11^d^	1.56 ± 0.04^e^	0.44 ± 0.02^e^	0.92 ± 0.06^e^	1.67 ± 0.07^f^	0.96 ± 0.52^a^	2.33 ± 0.52^de^	9.44 ± 1.34^f^	16.76 ± 0.00^f^	26.20 ± 0.03^e^

*Different letters in the same column indicate significant differences (p < 0.05).*

The total amino acids were divided into essential and semi-essential amino acid (EAA) and non-EAA groups. It is found that the EAAs and non-EAAs differ significantly between different sweet potato varieties. For sweet potato samples, SP02 (24.09 mg/g) and SP04 (8.17 mg/g) exhibited the highest and lowest EAA contents. The same was observed for the non-essential contents at 52.98 and 12.27 mg/g, respectively. Ju et al. (32) observed that sweet potato variety of Jishu 4 had the highest EAA content at 12.39 mg/g, while a variety of Xushu 18 had the lowest EAA content at 6.24 mg/g. This indicated that the amino acid composition of SP02 was relatively balanced, and the protein content level was high.

The nutritional values of sweet potatoes were further evaluated by the limiting amino acids (32). Among the seven sweet potato varieties, the limiting amino acids are cysteine (Cys) and methionine (Met). The amino acid limiting the biological value of sweet potato protein was mainly lysine, followed by arginine and methionine (1). The difference in the results is due to the different genotypes. The average contents of the Cys and Met were 0.64 and 0.71 mg/g, respectively, of which SP05 has maximum Cys content (0.71 mg/g). In addition to these two restricted amino acids, the contents of tyrosine (Tyr) and histidine (His) are also low, and the average values were 0.98 and 0.81 mg/g, respectively.

### Mineral Contents

The content of 12 mineral elements was determined. A large number of elements were K and P, and the medium elements were Mg and Ca, and the trace elements were Fe, Zn, Cu, Mn, Cr, Ni, Se, and Cd (Table 4). The average content of minerals of these seven sweet potatoes was in the order K > P > Ca > Mg > Mn > Fe > Zn > Cu > Ni > Se > Cr > Cd. In detail, the average content of K was 10,641 mg/kg with a range of 5885.50–18,286 mg/kg. The mean of P was 1343.3 mg/kg with a range of 803.6–2158.1 mg/kg. The content of K was about 2.73–22.75 times of P content. The average content of Mg was 566.32 mg/kg, ranging from 301.45 to 928.05 mg/kg. The average content of Ca was 738.8 mg/kg, and the amplitude of change was 401.25–1260.35 mg/kg. Among the trace elements, the contents of Mn, Fe, Zn, and Cu were relatively high, with the average contents of 23.79, 15.79, 10.42, and 6.26 mg/kg, respectively; the contents of Ni, Se, Cr, and Cd were relatively low, with average levels of 1.19, 0.049, 0.040, and 0.020 mg/kg. Therefore, sweet potato was a promising flour for supplementary claims in the nutraceutical and pharmaceutical industries (8). However, our results were inconsistent with Ju et al. (32), who obtained that the most abundant macro-element was K (196.39–531.29 mg 100 g^–1^ DW) and Ca (190.70–405.82 mg 100 g^–1^ DW) in ten sweet potato cultivars.

**TABLE 4:** The mineral content of sweet potato powder with different varieties.

	Ca (mg/kg)	Cu (mg/kg)	Fe (mg/kg)	K (mg/kg)	Mg (mg/kg)	Mn (mg/kg)	P (mg/kg)	Zn (mg/kg)	Cr (mg/kg)	Ni (mg/kg)	Se (mg/kg)	Cd (mg/kg)
SP01	401.25^f^	7.60^b^	14.80^d^	8730.5^f^	484.30^d^	9.10^e^	1068.05^e^	8.55^d^	0.038^a^	1.37^c^	0.043^c^	0.007^e^
SP02	1260.35^a^	8.85^a^	20.75^a^	18286^a^	802.15^b^	96.00^a^	1434.30^c^	16.15^a^	0.043^a^	1.60^a^	0.062^a^	0.032^b^
SP03	172.65^g^	2.15^f^	10.25^f^	11699^b^	301.45^g^	14.20^c^	803.60^f^	10.20^b^	0.043^a^	1.06^d^	0.043^c^	0.018^c^
SP04	782.60^d^	5.25^e^	13.30^e^	9971.5^d^	364.08^f^	15.95^b^	861.05^f^	8.35^d^	0.028^b^	0.77^f^	0.053^b^	0.021^c^
SP05	446.60^e^	6.85^c^	17.05^c^	10558.5^c^	432.85^e^	16.10^b^	1259.15^d^	10.20^b^	0.042^a^	1.51^b^	0.051^b^	0.01^de^
SP06	1131.90^b^	6.55^d^	19.25^b^	9353.5^e^	928.05^a^	4.95^f^	2158.10^a^	9.60^c^	0.043^a^	1.08^d^	0.052^b^	0.013^d^
SP07	976.25^c^	6.55^d^	15.15^d^	5885.5^g^	651.35^c^	10.20^d^	1818.65^b^	9.90^bc^	0.032^b^	0.93^e^	0.036^d^	0.040^a^

*Different letters in the same column indicate significant differences (p < 0.05).*

### Pasting Viscosity Characteristics

The viscosity characteristics of seven sweet potato flours are shown in Table 5. The range of PV was 90.7–318.8 RVU, where SP06 had the lowest PV value, and SP04 had the highest PV value. Aina et al. (33) obtained the PV with 9.6–100.2 RVU, which could be attributed to different drying methods. Air drying in an oven as carried out in the study of Aina et al. (33) might cause starch degradation, leading to low PV. The PV was reported to correlate negatively with the AC of the starch in flours. Amylose affected the swelling capacity of starch by restricting it and hence lowering the PV (34). It was generally observed that flours with high AC also showed low PV in these samples (27).

**TABLE 5:** Pasting properties and textural properties of different varieties of sweet potato powder.

	PV (RVU)	HPV (RVU)	BD (RVU)	CPV (RVU)	SB (RVU)	PT (°C)	HD (g)	Coh
SP01	285.7 ± 7.4^b^	193.8 ± 4.3^b^	91.9 ± 3.1^b^	251.3 ± 3.4^b^	57.5 ± 0.9^ab^	80.3 ± 0.5^bc^	18.5 ± 0.3^a^	0.5 ± 0.0^c^
SP02	167.0 ± 1.4^f^	139.6 ± 0.2^e^	27.5 ± 1.5^e^	175.4 ± 0.6^e^	35.9 ± 0.4^c^	82.8 ± 0.6^ab^	9.7 ± 0.5^d^	0.3 ± 0.0^d^
SP03	244.8 ± 3.2^d^	180.3 ± 2.8^c^	64.4 ± 0.3^c^	223.6 ± 6.2^c^	43.3 ± 3.4^c^	79.6 ± 0.6^bcd^	11.8 ± 0.3^c^	0.2 ± 0.1^e^
SP04	318.8 ± 0.7^a^	208.3 ± 0.1^a^	110.5 ± 0.8^a^	272.7 ± 2.4^a^	64.4 ± 2.3^a^	84.8 ± 3.4^a^	18.3 ± 0.8^a^	0.5 ± 0.0^c^
SP05	216.5 ± 1.8^e^	183.3 ± 1.9^c^	33.3 ± 3.7^d^	222.8 ± 4.0^c^	39.5 ± 2.1^c^	76.3 ± 0.6^d^	9.5 ± 0.2^d^	0.7 ± 0.0^a^
SP06	90.7 ± 0.2^g^	77.3 ± 0.5^f^	13.5 ± 0.7^f^	102.6 ± 3.1^f^	25.3 ± 2.7^d^	79.0 ± 0.2^cd^	8.2 ± 0.6^e^	0.6 ± 0.0^b^
SP07	261.8 ± 2.2^c^	153.5 ± 3.8^d^	108.3 ± 1.7^a^	206.2 ± 10.8^d^	52.7 ± 7.0^b^	80.8 ± 1.2^bc^	12.7 ± 0.0^b^	0.3 ± 0.0^e^

*BD, breakdown; CPV, cool paste viscosity; HPV, hot paste viscosity; PT, pasting temperature; PV, peak viscosity; SB, setback; HD, hardness; Coh, cohesiveness. Data are presented as mean ± standard deviation (SD). Different letters in the same column indicate significant differences (p < 0.05).*

Hot paste viscosity (HPV) is the minimum viscosity value in the constant temperature phase of the RVA profile measuring the ability of paste to withstand breakdown during cooling (9). Here, the HPV ranged from 77.3 (SP06) to 208.3 RVU (SP04). Fetuga et al. (12) reported 4.3–140.0 RVU of HPV for sweet potato flour prepared from different varieties and drying methods. Olatunde et al. (25) reported a range of 7.1–145.8 RVU of HPV for sweet potato flours prepared from different varieties, pretreatments, and drying methods. High HPV values may represent low cooking losses and the superior eating quality (35).

Cold paste viscosity (CPV) indicates the ability of a material to gel or paste after cooking or cooling in actual use (36) and determines a particular starch-based food quality (9). The range of CPV values was 102.6–272.7 RVU, the variety with the highest CPV value was SP04, and the variety with the lowest CPV value was SP06. Olatunde et al. (25) reported a range of 10.2–225.5 RVU of CPV for sweet potato flours prepared from different varieties, pretreatments, and drying methods. A high value of CPV has been attributed to the aggregation of amylose and a low final viscosity indicates the resistance of the paste to shear stress during stirring (35). The CPV of sweet potato flour from various varieties and at different processing methods has been collated by Fetuga et al. (12) and the values vary considerably not only among varieties but also at different processing methods.

Breakdown viscosity (BD) is an indicator of the paste’s resistance to disintegration in response to heat and shear (9). It has occurred as a result of holding slurries at high temperature and the difference between the PV and the trough viscosity (37). In this study, the range of BD values was 13.5–110.5 RVU. Fetuga et al. (12) found that BD has ranged from 0.5 RVU of American orange-fleshed to 92.3 RVU of Nigerian yellow-fleshed as compared to 34.9 RVU of Ugandan yellow-fleshed varieties. The BD in this study is similar to that reported by Olatunde et al. (25) who reported it in the range of 11.0–125.3 RVU but higher than that reported by Aina et al. (33) who reported it in the range of 3.3–50.2 RVU. A lower breakdown value of the sample indicated higher stability of starch under thermal conditions (9, 36) and higher stability of its paste (38).

### Gel Textural Properties

The texture characteristics of sweet potato powder are listed in Table 5. The HD value was in the range of 8.20–18.48 g, among which SP06 had the lowest HD value, and the HD value in SP01 was the highest. The range of Coh was from 0.22 (SP03) to 0.68 (SP05). In this experiment, the difference in Coh was very large. This may be caused by the differences in other compounds in different sweet potato whole powders, such as protein and cellulose. It showed that there were both high-cohesive varieties and low-cohesive varieties among the seven types of sweet potatoes.

### Gelatinization Properties

According to the gelatinization endothermic peak in the DSC curve, the gelatinization onset temperature (To), peak temperature (Tp), conclusion temperature (Tc), and gelatinization enthalpy (ΔH) can be calculated. Initial temperatures were closed to those obtained by Giselle et al. (39), which ranged from 59.39 (SP06) to 71.91°C (SP04), while Tp was higher than those obtained by Guo et al. (40), between 70.19 (SP01) and 88.40°C (SP07). The range of Tc value was 78.98–95.79°C, higher than those found by Ndangui et al. (30). SP02 had the highest Tc value, and SP03 had the lowest Tc value (Table 6). Varietal differences, environmental conditions, and the experimental protocols, such as the level of moisture, sample preparation, rate of heating, and instrument, were the factors affecting the gelatinization temperatures values (41). The lower values of enthalpy were noticed in this study, as compared to those reported by Ndangui et al. (30). The range of ΔHg was 1.85–5.65 J/g. This indicated that lesser energy was needed to break the intermolecular bonds in starch granules of such flour to achieve gelatinization (38).

**TABLE 6:** Gelatinization properties of different varieties of sweet potato powder.

	To (°C)	Tp (°C)	Tc (°C)	ΔH_g_ (J/g)
SP01	66.66^ab^	70.19^d^	94.19^a^	5.58^a^
SP02	61.95^ab^	73.02^cd^	95.79^a^	4.27^ab^
SP03	65.11^ab^	76.27^c^	78.98^e^	4.99^a^
SP04	71.91^a^	81.04^b^	89.83^b^	4.74^a^
SP05	60.77^ab^	81.08^b^	81.64^d^	3.62^ab^
SP06	59.39^b^	83.12^b^	85.68^c^	1.85^b^
SP07	68.99^ab^	88.40^a^	91.51^b^	5.65^a^

*Tc, conclusion temperature; To, onset temperature; Tp, peak temperature; ΔHg, enthalpy of gelatinization. Different letters in the same column indicate significant differences (p < 0.05).*

## Conclusion

In this study, the nutrition quality and physicochemical properties (viscosity, texture, and gelatinization) of seven sweet potato flours were analyzed for food processing. Results showed that the SP05 had the highest phenolic acid content and SP06 had the highest antioxidant activity among these varieties, reflecting the genetic diversity of sweet potato in phenolic substances. However, SP02 had the highest content of total protein, total amino acids, and EAAs, while SP05 and SP03 had the highest content of two limiting amino acids (Cys and Met). The K and P were the two most abundant elements of sweet potatoes, which was positively correlated with each mineral. The starch content of SP01 and SP04 was higher than 60% and had higher viscosity, which may have wide application in food processing. These results provided a theoretical basis for the selection of suitable sweet potato flour for food processing.

## Data Availability Statement

The original contributions presented in this study are included in the article/supplementary material, further inquiries can be directed to the corresponding authors.

## Author Contributions

JB and CT contributed to the conception and design of the study. LZ, YG, BD, and WR performed the experiments and analyzed the data. LZ and CT wrote the first draft of the manuscript. YG and JB wrote sections of the manuscript. All authors contributed to manuscript revision, read, and approved the submitted version.

## Conflict of Interest

The authors declare that the research was conducted in the absence of any commercial or financial relationships that could be construed as a potential conflict of interest.

## Publisher’s Note

All claims expressed in this article are solely those of the authors and do not necessarily represent those of their affiliated organizations, or those of the publisher, the editors and the reviewers. Any product that may be evaluated in this article, or claim that may be made by its manufacturer, is not guaranteed or endorsed by the publisher.
